# Fruit‐Penetrating Ability is Associated With the Number of Modified Lateral Bristles in the Ovipositor of *Drosophila suzukii*

**DOI:** 10.1002/ece3.72107

**Published:** 2025-09-19

**Authors:** Madelein Sara Micaela Ortiz, L. Gandini, M. C. Sabio, L. E. Bennardo, L. M. Matzkin, E. Hasson, J. Hurtado

**Affiliations:** ^1^ Departamento de Ecología, Genética y Evolución ‐ IEGEBA (UBA‐CONICET), Facultad de Ciencias Exactas y Naturales Universidad de Buenos Aires Ciudad Autónoma de Buenos Aires Argentina; ^2^ Department of Entomology University of Arizona Tucson Arizona USA; ^3^ Instituto de Fisiología, Biología Molecular y Neurociencias (UBA‐CONICET), Facultad de Ciencias Exactas y Naturales Universidad de Buenos Aires Ciudad Autónoma de Buenos Aires Argentina

**Keywords:** artificial selection, heritability, morphological adaptations, spotted wing drosophila, thorn bristles

## Abstract

In some insects, the evolution of herbivory was facilitated by the acquisition of specialized structures that confer adaptive advantages, allowing access to new ecological niches. A notable evolutionary innovation in *Drosophila suzukii* is the ovipositor, characterized by an arrangement of heavily sclerotized bristles aligned along the distal margins of the plates. This structure allows flies to pierce the skin of fruits, facilitating oviposition inside the fruit and consequently annulling the agricultural value of the fruit. However, the specific features of the ovipositor that underlie the ability to penetrate the fruit remain poorly understood. To address this gap, we investigated variation in the number of modified lateral bristles (MLBs) and assessed whether this trait may affect the ability to penetrate the fruit skin during oviposition. Our study revealed remarkable intrapopulation variation, with a substantial proportion of the genetic variance being additive. We also performed selection experiments and were able to both increase and decrease the number of MLBs. Finally, oviposition assays showed that females with more MLBs produce more perforations in the skin of blueberries. Overall, these results suggest that MLBs may contribute to fruit‐piercing ability during oviposition, and the substantial amount of additive genetic variance indicates that the number of MLBs can evolve. We highlight the importance of further studies to shed light on the subtleties of the genetic architecture of the trait.

## Graphical abstract

MLB number of *D. suzukii*'s ovipositor is heritable and responds to artificial selection. MLB number is plastic to the rearing temperature and it plays a key role in the ability of *D. suzukii* to pierce the skin of blueberries.

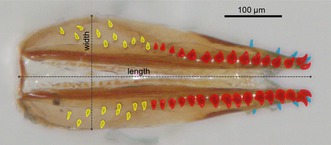

## 1 Introduction

The animal kingdom has plenty of surprising morphological adaptations that illustrate how species evolved to conquer new niches. From the various beak shapes of Darwin's finches (Grant 1999) to the prehensile tails of New World monkeys (Youlatos 1999), these adaptations demonstrate the power of natural selection to drive change in species to facilitate the exploitation of previously inaccessible resources and habitats. Among invertebrates, a fascinating example is the serrated ovipositor of the spotted wing drosophila, *Drosophila suzukii*, a fly native to Southeast Asia that has become a worldwide agricultural pest (Garcia et al. 2022).

Unlike most *Drosophila* species that utilize decaying fruits and other organic matter, *D. suzukii* possesses a specialized ovipositor adapted to penetrate the skin of intact ripe fruits (Rota‐Stabelli et al. 2013). This adaptation features a robust row of conical, enlarged, and sclerotized bristles along the distal margin of each ovipositor plate. These conical modified bristles are thought to enable females to lay eggs directly inside the intact fruit, allowing larvae to bypass external plant defenses and other threats by hatching inside the fruit. The ability to exploit healthy ripening soft fruits, such as blueberries, endowed *D. suzukii* with a reproductive advantage that likely contributed to its global spread as an agricultural pest (Atallah et al. 2014). This behavior also increases the risk of secondary infections in host fruits, resulting in significant economic losses (Rota‐Stabelli et al. 2013; Cini et al. 2012).

In addition to *D. suzukii*, its sister species, *D. subpulchrella*, is the only other member of the genus with a distinctively long, serrated ovipositor capable of puncturing intact fruit skin, albeit less efficiently (Atallah et al. 2014). The evolution of this fruit‐penetrating ovipositor involved significant modifications, including enlargement and sclerotization of specific conical bristles, as well as an increase in the number of conical bristles and overall size of the ovipositor (Atallah et al. 2014). These changes likely arose before the divergence of these sister species, 2–3 Mya (Suvorov et al. 2021) and after the split from its closest relative, *D. biarmipes*, 6–9 Mya (Ometto et al. 2013), which lacks a serrated ovipositor.

Similar evolutionary innovations occurred in the leaf‐mining species of the genus *Scaptomiza* during the transition to herbivory ~10 Mya (Aguilar et al. 2024). These flies, which target Brassicaceae crop leaves rather than fruits, possess strikingly similar ovipositors to those of *D. suzukii*. Such resemblance suggests parallel evolution, potentially underpinned by shared genetic mechanisms. Moreover, densely toothed, plant‐penetrating ovipositors—though not always morphologically identical to those of *D. suzukii*—have independently evolved several times within the Drosophilidae family, consistently associated with transitions to herbivory (Peláez et al. 2022). These patterns suggest that modified conical ovipositor bristles are highly amenable to adaptive evolution.

Recent studies on fruit borers have shown that the physicochemical properties of host fruits—such as increasing hardness during fruit development—can significantly deter insect feeding and damage (Zhang et al. 2024). In this light, it is plausible that similar selective pressures imposed by fruit features shaped the evolution of the ovipositor in *D. suzukii*. The arrangement, number, size, and rigidity of its conical bristles may have evolved in response to the physicochemical challenges posed by different fruit characteristics. Thus, plant traits like skin firmness could have been important drivers in the coevolutionary arms race between *D. suzukii* and its hosts, acting in tandem with morphological innovations that enhance fruit penetration.

**FIGURE 1:**
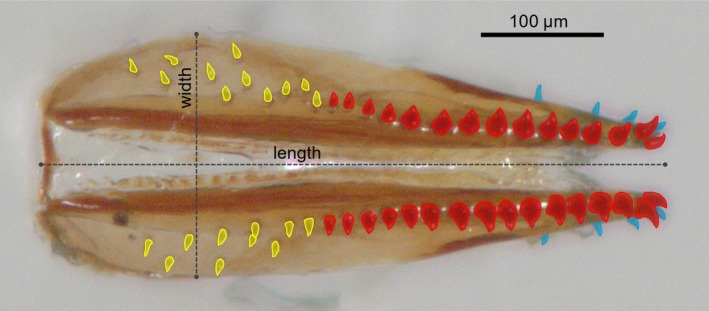
Postero‐anterior view of a serrated ovipositor (100×), showing the MMBs in blue, ULBs in yellow and MLBs, according to the criteria described in Section 2, in red.

Ovipositor conical bristles are mono‐innervated and play a role in mechanotransduction, likely sensing substrate stiffness and penetration success during egg laying (Crava et al. 2020; Dweck et al. 2021). These bristles can be categorized into three types based on their structure and location: modified lateral bristles (MLBs, also known as modified conical pegs type 1 or thorn bristles type 1), unmodified lateral bristles (ULBs, also known as thorn bristle type 2) and modified marginal bristles (MMBs, also known as conical pegs type 2 or thorn bristles type 2) (Atallah et al. 2014; Crava et al. 2020; Dweck et al. 2021). MLBs, which cover approximately half the length of the ovipositor and give the organ its saw‐like appearance, are tightly aligned along the lateral margins of the ovipositor plates. Apical MLBs are larger and more sclerotized than their proximal counterparts. ULBs are smaller and less stout and appear dispersed further from the lateral margins. In contrast, MMBs are confined to the apical region and are aligned along the medial line of each plate. The MLBs and MMBs are the primary structures that come into contact and directly interact with fruit skin during penetration (Figure 1). While these bristles are presumed to confer the ability to penetrate fruit, this causal relationship remains unverified.

Several physical characteristics of these modified structures—such as bristle length and rigidity—may play important functional roles in piercing ability. In this study, however, we focus on the number of modified bristles as an initial step toward understanding the structural adaptations of the ovipositor related to fruit penetration. Notably, the number of MLBs varies within *D. suzukii* populations, whereas the number of MMBs remains constant at four (Atallah et al. 2014; Crava et al. 2020; Ortiz et al., personal observation). However, the connection between the number of MLBs and the fruit‐penetrating ability has yet to be conclusively established. The present study aims to investigate the genetic basis of variation in the number of MLBs, assess plasticity in response to temperature, and evaluate how the MLBs affect the ability of females to penetrate blueberry skin during oviposition.

## 2 Materials and Methods

### 2.1 Fly Stocks

Lines used in this paper were derived from three collecting sites in Argentina: Mercedes, Luján, and Chubut, which we consider to represent three distinct populations. Adult flies were collected with entomological nets from plastic buckets containing mashed banana fermented with yeast, strategically placed along transects in each site. Flies were initially housed in bottles containing standard cornmeal medium (medium from here on) for 6–24 h. Subsequently, females were individually transferred to vials with fresh medium to generate isofemale lines (lines from here on). Lines were classified as *D. suzukii* by the presence of spotted wings on adult male progeny.

One hundred and twenty lines were founded from flies collected near a blueberry orchard in Mercedes, Buenos Aires Province, Argentina (34°38′32.5′′ S, 59°23′36.6′′ W) in 2023. Sixteen lines were founded from flies collected on a plum orchard in Luján, Buenos Aires Province (34°35′05.5′′ S, 59°04′40.6′′ W) in 2020 (Gandini et al. 2023). Twenty‐four lines derived from collections near a raspberry orchard in El Bolsón, Chubut province (42°1′56′′ S, 71°31′58′′ W) in 2022 and were kindly provided by J. Mensch.

All established lines were maintained in the lab for at least four generations before conducting the experiments described below. Unless otherwise specified, all experiments in this paper were performed at 25°C, and flies were reared at the same temperature under a 12:12 light–dark cycle.

### 2.2 Phenotypic Characterization and Survey of MLB Variation

The distinction between modified and unmodified lateral bristles is often unclear (Akutsu and Matsuo 2023). Therefore, we define the number of MLBs—the phenotypic trait we used to characterize variation in ovipositor morphology—as the number of conical bristles along the distal margin of the ovipositor, starting at the most distal bristle and continuing until reaching the first bristle for which the space to the next one exceeds its diameter. Bristles not meeting this criterion were not counted (Figure 1). This approach enabled us to focus specifically on the bristles at the margins of the ovipositor plates since we presume they are specifically contributing to the ability to pierce the fruit's skin. To characterize the number of MLBs, we randomly selected 15 lines per population.

We examined the ovipositors of 900 cold‐sacrificed females (20 per line [×15] per population [×3]) under a stereomicroscope at 40× magnification. We recorded the number of MLBs from both the left and right plates. Then, for each ovipositor, we calculated the between‐sides mean value and, as a proxy for bilateral fluctuating asymmetry, the absolute difference between sides (Palmer and Strobeck 1992).

To evaluate differences between populations, we fitted two generalized linear mixed models using the *glmmTMB* function of the *glmmTMB* package (Brooks et al. 2017). One model was for assessing the mean number of MLBs averaged between sides (MLB number from here on), and the other was for examining MLB fluctuating asymmetry (absolute difference between sides). The mean MLB model had a normal error structure, and the asymmetry one had a Conway‐Maxwell‐Poisson (compois) error structure. In both cases, Population was treated as a fixed factor and Line as a random factor nested in Population. The significance of the Population effect in each model was determined using a Type II Wald test with the *car* package (Fox et al. 2019). Post hoc pairwise comparisons between populations were performed using the *emmeans* function of the *emmeans* package (Lenth et al. 2024).

To explore differences in the number of MLBs between lines within each population, we ran two additional GLMMs with normal error structures per population. One model included Line as a random factor, while the other was an intercept‐only model (without Line). To assess the significance of Line in each population, we compared the goodness of fit between these two models by conducting a likelihood ratio test using the *anova* function of the *stats* package (R Core Team 2024). The same approach was implemented to explore fluctuating asymmetry differences between lines in each population.

For all analyses presented in this study—including those described in the subsequent subsections—models fit and assumptions were evaluated using residual simulations with the *simulateResiduals* function of the *DHARMa* package (Hartig et al. 2020). All statistical analyses in the paper were conducted in R v4.3.3 (R Core Team 2024), and all results were visualized using the *ggplot2* package (Wickham 2016).

### 2.3 Phenotypic Plasticity

Phenotypic responses to rearing temperature were evaluated in four randomly chosen lines. Each line was subjected to two rearing temperatures, 17°C and 25°C, during the whole life cycle for four generations before phenotypically characterizing their ovipositors. The MLB number and asymmetry were determined in 40 adult females per line and thermal treatment.

For MLB number, a GLMM with a normal error structure was fitted using the *glmmTMB* function of the *glmmTMB* package, including Line, Temperature, and Line by Temperature interaction as fixed factors. For MLB asymmetry, a GLMM was fitted using a negative binomial error structure (“nbinom2” option in *glmmTMB*), considering the same fixed factors. Significance was determined using a Wald type II test with the *car* package. Tukey post hoc pairwise comparisons were performed using the *emmeans* function of the *emmeans* package.

### 2.4 Estimation of MLB Number Heritability

A genetically variable experimental population was founded by crossing five males and five females from each of the 120 Mercedes lines in a mating cage in an “all‐against‐all” design. We maintained this population with at least 1000 adult individuals per generation for four generations before initiating the experiment. Starting from this population, which we denominated the base population, we estimated the heritability (*h* ^2^) of the number of MLBs using a half‐sib design, according to the methodology described in Falconer and Mackay (1996).

Firstly, fifteen 4‐ to 6‐day‐old adult males (Sires) were randomly selected, and each was crossed with four equally aged virgin females (Dams). Inseminated Dams were then placed individually in vials with fresh medium for oviposition. Finally, the number of MLBs was measured in 10 female offspring from each Dam, resulting in 40 phenotyped daughters per Sire.

To estimate *h* ^2^, we fitted a hierarchical linear mixed model using the *lme4* function of the *lme4* package (Bates 2015). The model included Sire and Dam (nested within Sire) as predictors of MLB number. Variance components (Sire, Dam, and residual) were extracted using the *VarCorr* function of the *lme4* package, and *h* ^2^ was estimated as four times the proportion of the variance explained by Sire (Falconer and Mackay 1996). The precision of the estimates was assessed by bootstrap analysis using the *boot* function of the *boot* package (Canty et al. 2024) to provide 95% confidence intervals from 1000 replicates. To further evaluate the significance of Sire's contribution, we ran a second model not including Sire as a predictor. We then compared the with‐Sire and without‐Sire models by performing a likelihood ratio analysis using the *anova* function of the *stats* package.

### 2.5 Artificial Selection

Starting from the above‐described base population, we applied an artificial selection protocol to generate divergent lines for the number of MLBs. The selection protocol consisted of two contrasting regimes: in one, which we called High, females with the highest MLB number scores were selected as progenitors for the next generation, and in the other, called Low, females with the lowest MLB number were chosen as progenitors. A non‐selection Control regime in which progenitors were randomly selected regardless of the number of MLBs was run in parallel to the selection lines. The protocol was replicated three times starting from the same base population.

In each generation and for each replicate, 150 females per regime were randomly selected, inseminated, and maintained in separate vials with fresh medium for oviposition. Each female was allowed to oviposit for five days. Next, females were separated and sacrificed for phenotypic characterization. In the High and Low regimes, only offspring from the 20 females with the most extreme phenotypes (either high or low, depending on the regime) were used as mothers for the next generation. In the Control regime, 20 inseminated females were randomly selected as mothers in each generation. After each bout of selection, an expansion generation without selection was implemented to increase the population size to at least 1000 adult individuals per regime. A total of ten generations, five generations of selection alternated with five expansion generations, were conducted. Phenotypic characterization of females was performed after each generation without selection.

To assess the effect of selection on MLB number, a GLMM with a normal error structure was fitted for each replicate using the *glmmTMB* package. Each model included Regime as a fixed factor with four levels: High, Low, Control, and G0. High, Low, and Control regimes refer to the last (10th) generation, while G0 stands for the base population at the beginning of the experiment. The significance of the Regime effect was determined using a Type II Wald test with the *car* package. Pairwise Tukey multiple comparison tests between regimes were performed using the *emmeans* function of the *emmeans* package.

### 2.6 Oviposition Assays on Blueberries

**FIGURE 2:**
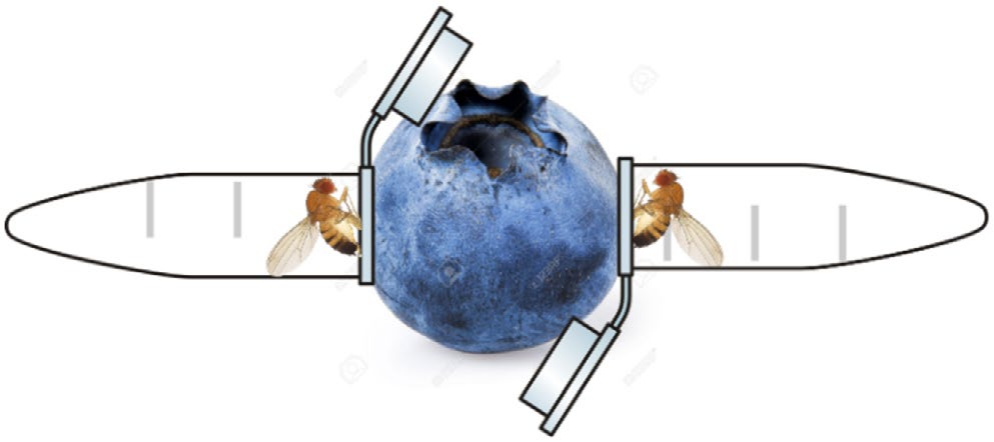
Schematic illustration of the experimental unit used to evaluate the relationship between the number of MLB and puncturing ability in *D. suzukii*. The illustration shows two females of different lines or treatments, with presumptively different MLB numbers, located on opposite sides of the same blueberry.

Groups of ten 4–6 days old virgin females were allowed to mate with 20 equally aged virgin males for 24 h in vials without medium, each containing a 6 cm × 12 cm piece of paper moistened with an 18% (m/v) fresh aqueous yeast suspension (m/v). This substrate provided both food and water while deterring oviposition. The inseminated females were briefly CO_2_‐anesthetized and placed individually in 1.7 mL Eppendorf tubes. To investigate the relationship between the number of MLBs and the ability of flies to pierce blueberry skin, females were tested for 4 h in pairs, with the two flies in each pair tested simultaneously on opposite sides of the same fruit (Figure 2).

To minimize the occurrence of pairs with no differences in MLB number, females in each pair were selected from three alternative sources of flies with presumably different numbers of MLBs: (1) divergent lines derived from field collections with distinguishable phenotypes, (2) flies reared at different temperatures (17°C and 25°C) and (3) divergent lines generated through the artificial selection regime. A total of 90 replicates were run for each source, resulting in 270 replicates in total.

After being assayed for 4 h, females were cold‐sacrificed and phenotyped for the number of MLBs as previously described, and blueberries were examined under a stereomicroscope at 40× magnification. The number of punctures on each blueberry side was recorded, counting both those with egg filaments protruding from the fruit skin and those without filaments. The number of eggs deposited beneath the fruit skin was also recorded. Fruits with zero punctures on both sides were excluded from the analysis.

The number of punctures was modeled with a GLMM with a Tweedie error structure using the *glmmTMB* package for the complete dataset and each source separately. The models included the number of MLBs as a numerical predictor and Blueberry (i.e., pairs of females) as a random factor. Blueberry was included as a random factor to account for fruit‐specific effects, as our goal was not to explore the influence of fruit traits per se, but to control for their potential impact. In addition, to assess the effect of the experimental condition, which was defined as line of origin in the first‐source trial, rearing temperature in the second‐source trial, and selection regime in the third‐source trial, we ran an additional set of GLMMs (also with Tweedie error structure) for each source separately. These models included Condition as a fixed factor and Blueberry as a random factor, along with the number of MLBs as a numerical predictor. Although interaction terms between Condition and MLB number were initially included, they were ultimately excluded from the final models because they did not improve model fit or show significant effects in any dataset. The significance of fixed effects, in these and all subsequent models, was determined using a Wald type II test with the *car* package.

To evaluate whether fruit ripeness influenced piercing ability, each blueberry used in the assays was classified into one of three ripeness categories based on internal color at the time of the assay (low: greenish; intermediate: yellow; high: reddish). Firmness was assessed as well, but it consistently matched the internal coloration (fruits with green interiors were firm, while those with red or purple interiors were soft). This allowed us to run a set of GLMs (also with Tweedie error structure) to model the number of punctures as a function of Ripeness (instead of Blueberry) and MLB number for each source separately. Given that Ripeness only had three levels, it was treated as a fixed factor. Although interaction terms between Ripeness and MLB number were initially included, they were ultimately excluded from the final models because they did not improve model fit or show significant effects in any dataset.

The association between the number of punctures and the number of eggs laid under the fruit skin was examined by Pearson correlation analyses using the *cor.test* function of the *stats* package.

To evaluate whether the number of MLBs correlates with ovipositor size, we measured ovipositor length and width alongside MLB number in an additional 40 females from the same line used in the second‐source oviposition trial. Images were captured using a Leica M205 A stereomicroscope and analyzed with the measurement tools of the Leica Application Suite software to determine ovipositor length and width. The length was defined as the distance between the ovipositor base and its distal tip along the central axis between the valves, while the width was measured at the widest part of the organ on a line perpendicular to the central axis (Figure 1). A Pearson correlation analysis was performed separately for each rearing temperature (Condition) using the *cor.test* function of the *stats* package. To further evaluate whether ovipositor dimensions serve as predictors of MLB number, we fitted two GLMs with a Gaussian error structure using the *glmmTMB* function of the *glmmTMB* package. One model included ovipositor length and its interaction with Condition as fixed effects, while the other included ovipositor width and its interaction with Condition.

## 3 Results

### 3.1 The Number of MLBs Varies Between and Within Populations

**FIGURE 3:**
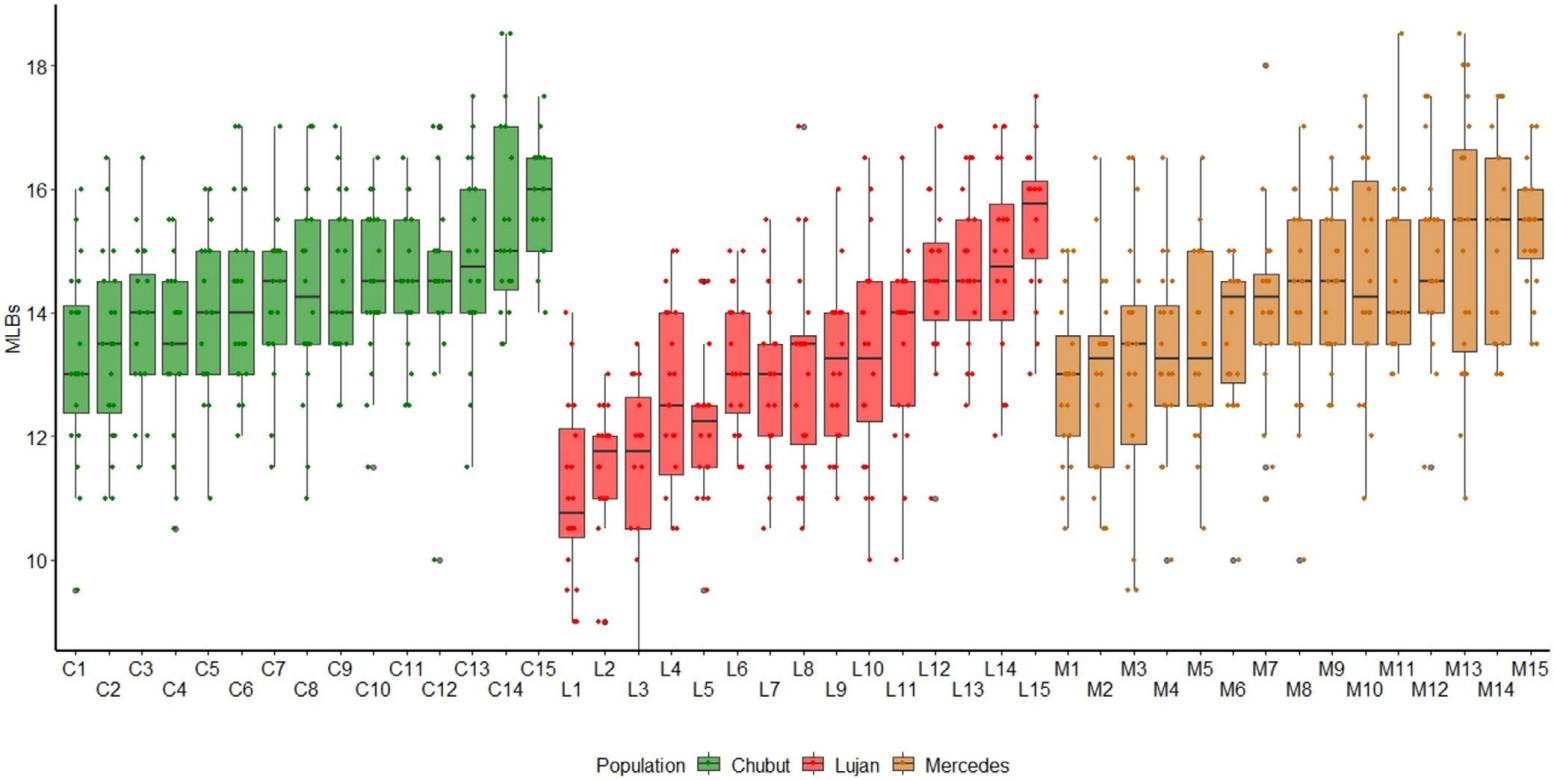
Number of MLBs in 15 lines of each one of three populations of *D. suzukii*: Chubut (green), Lujan (red) and Mercedes (dark orange). Outliers were excluded.

The number of MLBs varied between 9 and 18.5 among lines (after excluding outliers [0.9%]) and differed significantly between populations (*p* = 0.001) (Figure 3). Flies from Luján exhibited less modified bristles than those from Chubut (*p* = 0.002) and Mercedes (*p* = 0.013), whereas no differences were detected between the latter two (*p* = 0.861). After excluding outliers, MLB number varied from 9 to 17.5 in Luján, 9.5 to 18.5 in Mercedes, and 9.5 to 18.5 in Chubut. In addition to variation between populations, MLB number varied significantly between lines within each population (*p* < 0.001 in all populations). The analysis of components of variance showed that 37.54%, 17.64%, and 15.7% of total variation can be accounted for by differences among lines in Luján, Mercedes, and Chubut, respectively. These results suggest that trait variation has a genetic basis. Regarding asymmetry, no differences were found between populations (*p* = 0.846) or lines within populations (Mercedes: *p* = 0.478, Lujan and Chubut: *p* = 1).

### 3.2 The Number of MLBs Is Heritable

To confirm that variation has a genetic basis, we estimated the MLB number heritability using a half‐sib approach. We obtained a *h* ^2^ estimate of 0.47 (95% CI: 0.119–0.835), which indicates that additive genetic variance accounts for a substantial proportion of phenotypic variation. Also, the sire factor contributed significantly to the model's goodness of fit, confirming the existence of a non‐null variance component explained by sire. These results indicate that a significant proportion of the phenotypic variation in the number of MLBs is due to genetic differences inherited from parents.

### 3.3 The Number of MLB Is Plastic to Temperature

**FIGURE 4:**
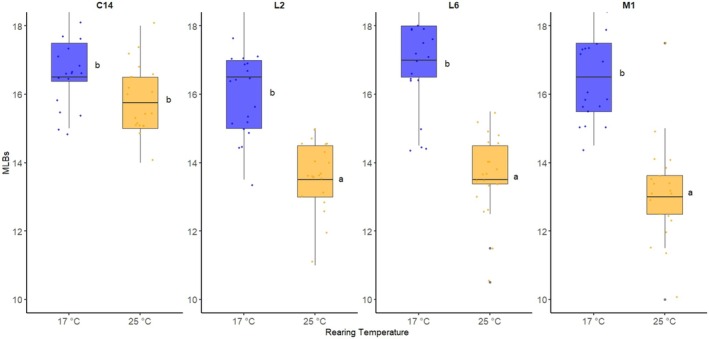
Number of MLBs of flies of four lines (L2, L6, C14, and M1) reared at 17°C and 25°C. The letter groupings indicate significant differences between groups (*p* < 0.05). Jitter was applied to the data points to reduce overplotting.

Considering that larval development is highly sensitive to thermal variation in *D. suzukii* (Jakobs et al. 2015), we evaluated the robustness of MLB number by comparing the ovipositors of flies reared at two thermal regimes: 17°C and 25°C. Females reared at 17°C had, on average, 2.6 more MLBs than those reared at 25°C (*p* < 0.001), which indicates a notable impact of rearing temperature on ovipositor morphology. The effect, however, depended on the line, as suggested by the significant Line × Temperature interaction (*p* < 0.001). This effect was particularly evident in line M1 (*p* < 0.001) and non‐significant in line C14 (*p* = 0.193) (Figure 4). In contrast, neither Temperature nor Line nor the Line × Temperature interaction affected asymmetry between ovipositor plates (*p* = 0.765, *p* = 0.230, or *p* = 0.465, respectively).

### 3.4 The Number of MLBs Responds to Artificial Selection

**FIGURE 5:**
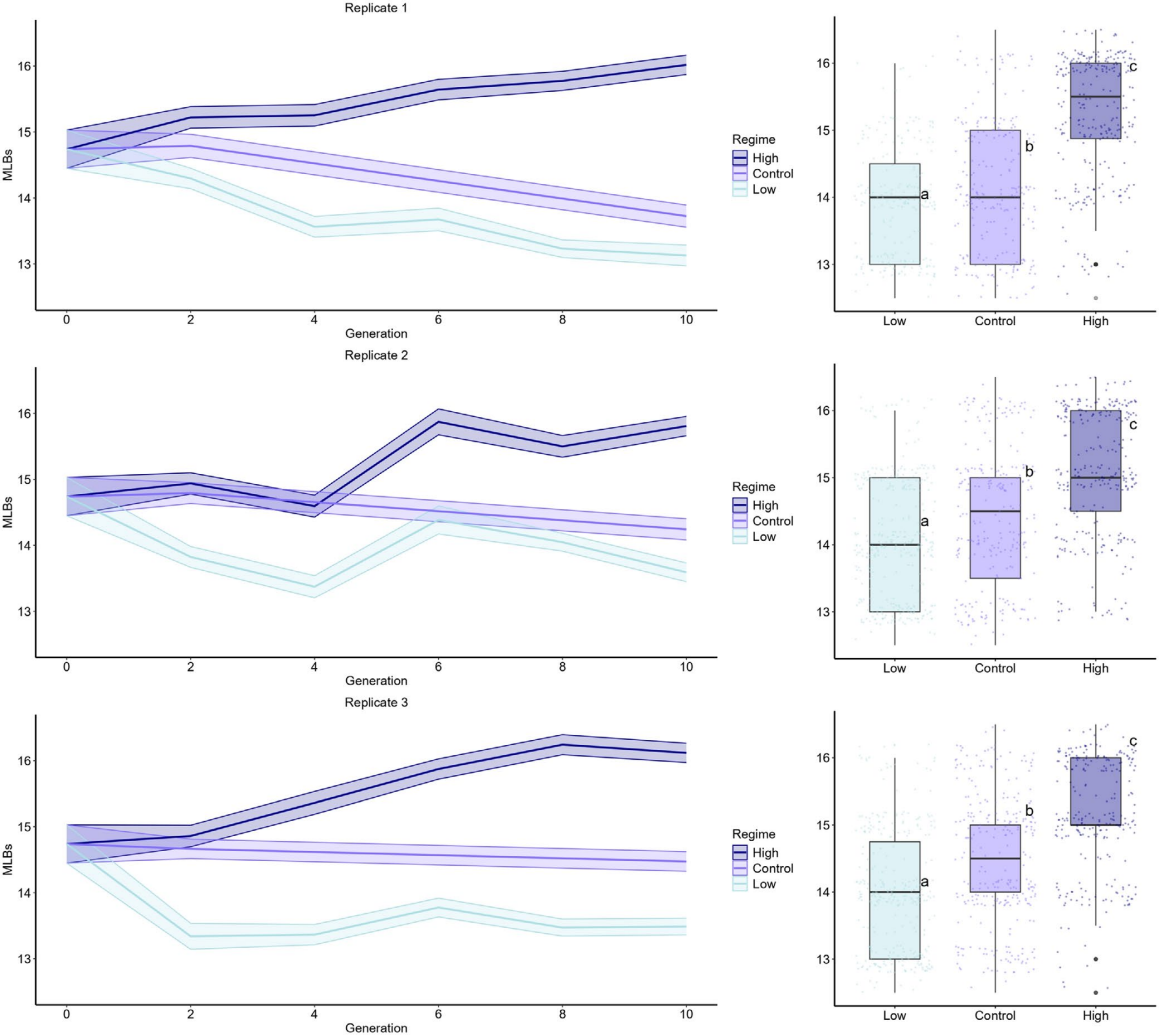
Evolutionary trajectory of the number of MLBs under selection regimes. Replicates 1, 2, and 3 represent three independent trials. On the left plots, lines represent the mean number of MLBs across generations, and envelopes represent the 95% confidence interval. Boxplots on the right show the number of MLBs per regime in the last (10th) generation, with letters indicating significant differences (Tukey's test, *p* < 0.05). Jitter was applied to the data points to reduce overplotting.

We further assessed the genetic basis of MLB number variation by performing artificial selection experiments in divergent directions (High and Low number of MLBs). For each selection regime and the control, we ran three independent replicates. After five generations of selection, the number of MLBs responded in both directions in all replicates (Figure 5). Interestingly, it showed a slightly decreasing trend throughout the experiment in the control regime. This was particularly true in one of the replicates in which the average number of MLBs significantly changed from 14.8 in G0 to 13.7 in G10 (*p* < 0.001), whereas G0 to G10 such decreases were not significant in the other two replicates (*p* = 0.14 and *p* = 0.6). This indicates moderate stability of the phenotype in the absence of artificial selection.

In contrast, the number of MLBs markedly differed between G0 and G10 in both selective regimes in all replicates (High G0 vs. G10: *p* < 0.001, Low G0 vs. G10: *p* < 0.001, for all replicates in both cases). In the High regime, the number of MLBs increased from an average across replicates of 14.8 in G0 to 16.0 in G10. In the Low regime, the change was more pronounced: MLB number averaged across replicates decreased from 14.8 to 13.4 in G10. Comparisons between selected and Control lines in the last generation also revealed significant differences in both selection regimes, confirming that selection was effective (High vs. Control: *p* < 0.001 for all replicates, Low vs. Control: *p* = 0.003 for one replicate and *p* < 0.001 for the other two).

### 3.5 The Number of MLBs Predicts the Number of Punctures on Blueberry Skin

To investigate the relationship between the number of MLBs and the ability of female flies to pierce blueberries, we selected pairs of females with presumptively different MLB numbers that were tested on opposite sides of the same fruit, as shown in Figure 2. The experiment was performed thrice, each time using alternative sources of flies. For the first‐source experiment, females in each pair came from lines C14 and M1 with divergent MLB number phenotypes. In the second‐source experiment, females proceeded from sets of M1 flies reared at 17°C and 25°C. In the third‐source experiment, females came from the High and Low selection regimes (10th generation, replicate 3).

**FIGURE 6:**
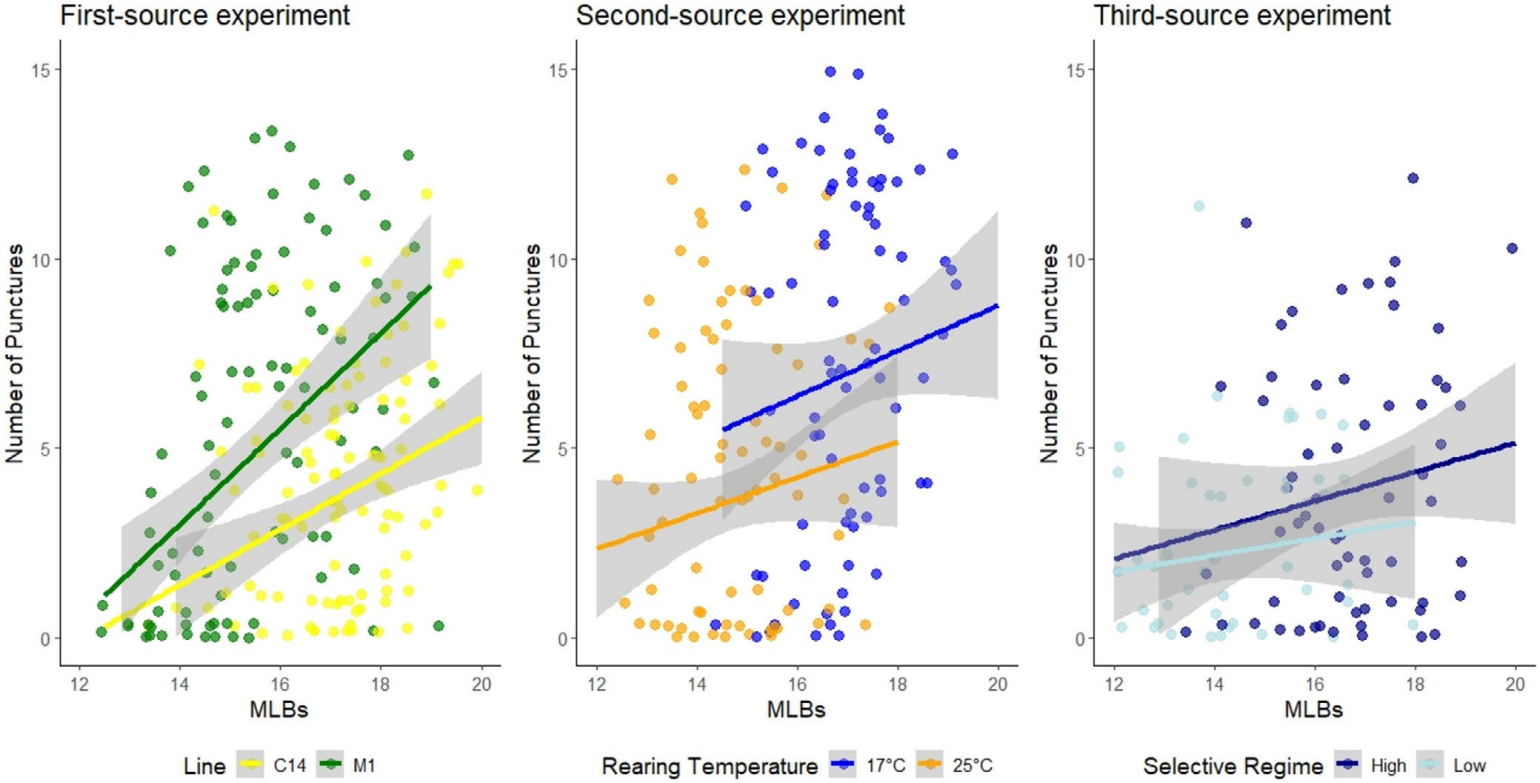
Relationship between MLB number and the number of punctures produced by *D. suzukii* female flies on the skin of blueberries. A regression line with its 95% confidence interval (gray envelope) is shown per line of thermal treatment in each experiment. As females were tested in pairs on opposite sides of the same fruit, each blueberry is represented by two dots. Jitter was applied to the data points to reduce overplotting.

Regardless of the flies' source, we found an overall positive relationship between the number of MLBs and the number of punctures in blueberry skin (Figure 6). Females produced, on average, 11.55% (*p* = 0.003), 22.71% (*p* < 0.001) and 14.66% (*p* = 0.002) more punctures per additional bristle in the first, second, and third‐source experiments, respectively. In addition, females reared at 17°C exhibited 2.6 more MLBs and performed 4.34 more punctures compared to those reared at 25°C in the second‐source experiment, on average. Similarly, in the third‐source experiment, high‐regime females had, on average, 2.7 more MLBs and made 1.9 more punctures than those of the low regime. However, it should be noted that C14 females—exhibiting, on average, 1.8 more MLBs—made, on average, 1.47 fewer punctures than those of the M1 line.

When Condition—defined as line of origin in the first‐source experiment, rearing temperature in the second‐source experiment, and selection regime in the third‐source experiment—was included as an explanatory variable, the number of MLBs remained a significant predictor of puncture count in both the first‐ and second‐source experiments (*p* < 0.001 and *p* = 0.008, respectively). Importantly, the magnitude of the MLB number effect remained similar after including Condition (21.49% and 13.48% increases in puncture number per additional bristle in the first‐ and second‐source experiment, respectively). This suggests that the relationship between MLB number and puncturing ability holds even within individual lines and temperature treatments. Condition itself also had a significant effect in both experiments (*p* < 0.001 and *p* = 0.020, respectively), pointing to the potential influence of additional morphological, physiological, or behavioral traits. In contrast, in the third‐source experiment (where flies originated from divergent lines generated through artificial selection), neither the number of MLBs nor Condition showed significant effects (*p* = 0.248 and *p* = 0.052, respectively).

When the Blueberry random factor was replaced by Ripeness (categorized as low, intermediate or high), MLB number also remained a significant predictor of puncture count across all three datasets (*p* = 0.002, *p* < 0.001 and *p* = 0.006 for the first‐, second‐ and third‐source experiments, respectively). In contrast, Ripeness itself had a significant effect only in the second dataset (*p* = 0.252, *p* < 0.001 and *p* = 0.233, respectively), where intermediately ripe fruits were more frequently pierced than those classified as low or high ripeness. This indicates that the influence of ripeness on puncturing ability was not consistent across trails.

**FIGURE 7:**
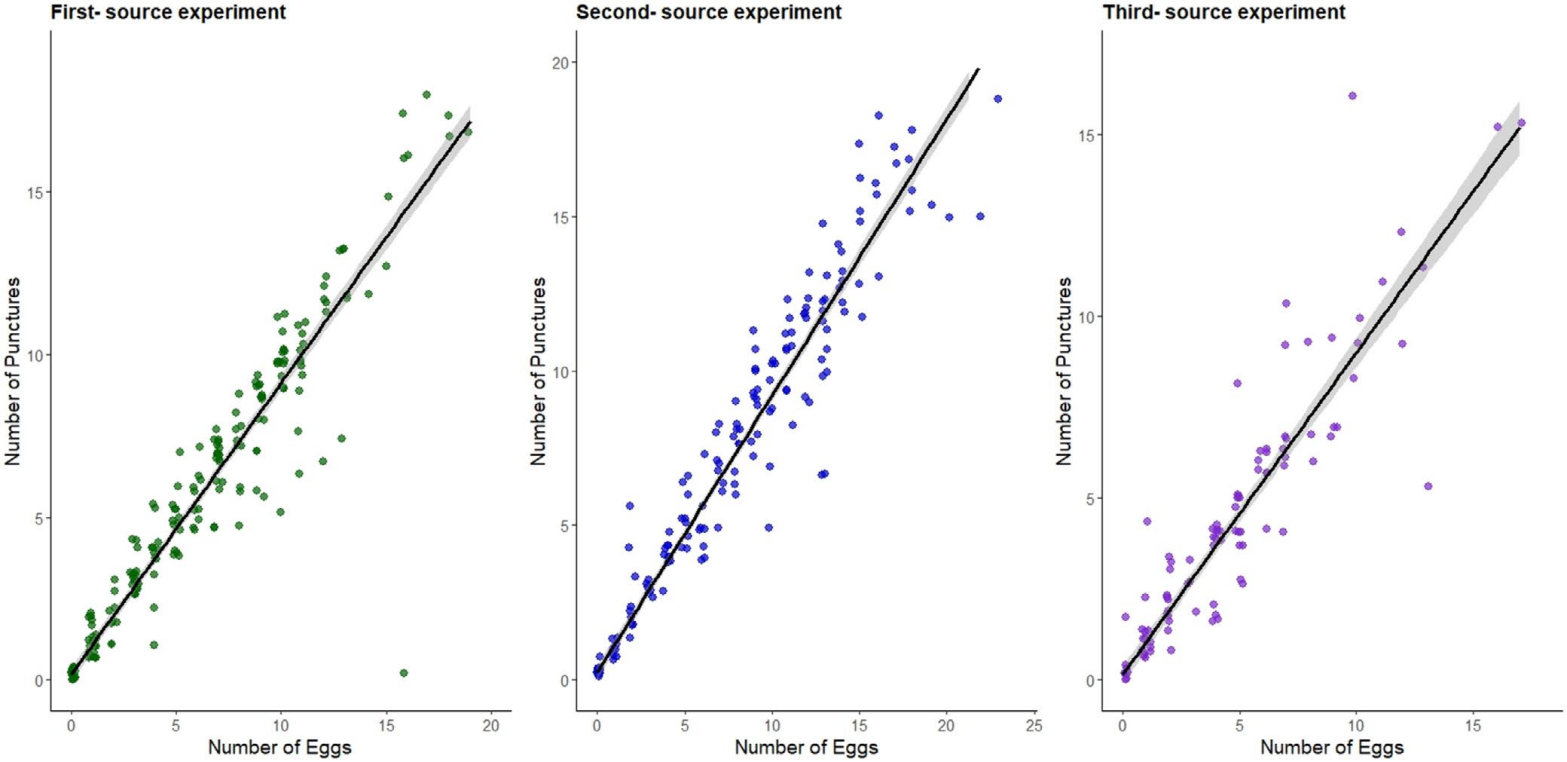
Relationship between the number of punctures and the number of eggs produced by *D. suzukii* female flies on the skin of blueberries. A regression line with its 95% confidence interval (gray envelope) is shown for each experiment.

Our analyses assessing the relationship between the number of punctures and the number of eggs laid beneath the fruit skin revealed a strong positive correlation between the two variables in the three datasets (*r* = 0.957, *r* = 0.973, *r* = 0.942, respectively; *p* < 0.001 in all cases) (Figure 7).

**FIGURE 8:**
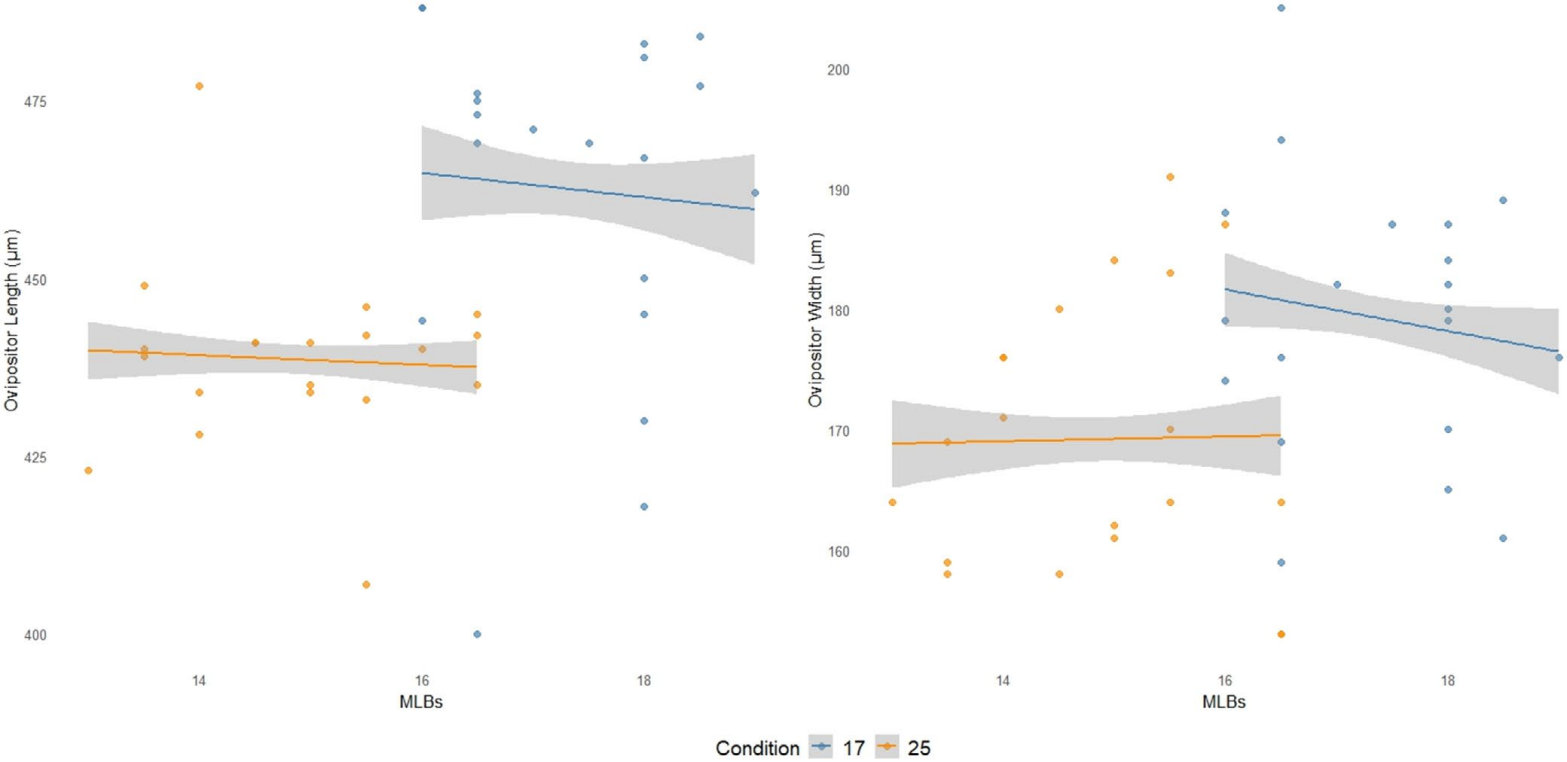
Relationship between the number of MLBs and ovipositor size (length and width, in μm) in *D. suzukii* females from M1 line reared at 17°C and 25°C. A regression line with its 95% confidence interval (gray envelope) is shown for each thermal treatment. Each dot represents a single female.

To examine the potential relationship between the number of MLBs and ovipositor size, we analyzed correlations between MLB number and ovipositor length, and between MLB number and ovipositor width, in 40 females from line M1, the same line used in the second‐source oviposition trial. Twenty of these females were reared at 17°C and 20 at 25°C. For flies reared at 17°C, we found no significant correlation between MLB number and either ovipositor length (*r* = −0.068, *p* = 0.776) or width (*r* = −0.147, *p* = 0.537) (Figure 8). Similarly, no correlation was found for flies reared at 25°C, with *r* = −0.057 (*p* = 0.812) for length and *r* = 0.019 (*p* = 0.937) for width (Figure 8). To further investigate this, we fitted two GLMs with MLB number as the response variable and either ovipositor length or width, along with rearing temperature (Condition), as predictors. In both models, Condition was the only significant explanatory variable (*p* < 0.001), while neither ovipositor length (*p* = 0.705) nor width (*p* = 0.713), nor their interactions with Condition (*p* = 0.917 and *p* = 0.615, respectively), had significant effects. These analyses suggest that MLB number has limited value as a proxy for ovipositor size within the same line and rearing temperature.

## 4 Discussion

In this study, we addressed the hypothesis that the number of modified lateral bristles (MLBs) of the serrated ovipositor affects fruit‐penetrating ability in the spotted wing fly *D. suzukii*, an agricultural fruit pest. We focused specifically on the lateral sclerotized bristles aligned along the distal margins of the plates. These modified bristles embody why the ovipositor is referred to as serrated, and we presume they can significantly contribute to the fruit‐damaging characteristic of this fly. To address this hypothesis, we investigated variation in the number of MLBs and its relationship with the number of punctures produced by females during oviposition in blueberries. We employed flies with different degrees of manipulation: isofemale lines, flies raised at different temperatures, and flies exposed to divergent selective regimes.

Differences between populations explained a small, though significant, proportion of total phenotypic variation (7.81%), while variation among lines within populations accounted for a larger proportion (25.4%). Notably, lines with the highest and lowest number of MLBs within each population displayed non‐overlapping ranges of variation (Figure 3), highlighting distinct morphological divergence at the intrapopulation level. These results concord with previous studies that reported differences between lines in the total number of bristles of the ovipositor in *Scaptomyza flava* (Peláez et al. 2022) and *D. suzukii* (Akutsu and Matsuo 2023). However, our study focuses specifically on the MLBs. Moreover, our study is the first to show that variation in the morphology of *D. suzukii* ovipositor has a genetic basis. This assertion is also supported by the results of our half‐sib experiment showing that additive genetic variance accounts for 47% of the total phenotypic variance and by the significant responses to divergent selection regimes.

During the last 18 years, *D. suzukii* has spread from its native range in East Asia and got established in North America, South America, Europe, and Africa (Little et al. 2020; Tait et al. 2021). In its invasive range, it dramatically expanded its fruit host range (Lee et al. 2011; Walsh et al. 2011). Thus, if the MLB number affects flies' ability to exploit certain fruits, the invasion of regions with new hosts may have been accompanied by the adaptive evolution of MLB number. In such a case, the evolutionary potential of the number of MLBs may have been determinant during the species' expansion. It is also possible that the genetic variation we observed within each studied population is maintained by divergent selection pressures imposed by host fruits with varying skin hardness. Future studies comparing patterns of MLB number variation in native and invasive populations exploiting different fruit hosts could shed light on the evolutionary mechanisms underlying the global spread of this pest.

Though the evolution of MLB number may allow flies to exploit new fruit hosts, it may be subsequently constrained by the effects that it likely exerts on the genital coupling during copulation (Akutsu and Matsuo 2023; Muto et al. 2018). Thus, it would be worthwhile to evaluate whether lines exposed to divergent selection pressures in the number of MLBs evolved differences in the duration of copulation, coupling time, or reproductive output. This could also help to evaluate whether a functional restriction imposed by sexual selection shaped the evolution of the number of MLBs.

*Drosophila subpulchrella*, the sister species of *D. suzukii*, has an ovipositor of similar size, also featuring modified bristles that allow piercing blueberry skin. However, this species is less efficient at puncturing the tougher skin of fruits such as grapes and has not expanded its distribution beyond East Asia (Atallah et al. 2014). Atallah et al. (2014) suggest that the morphological differences of the ovipositor between the species, ranging from a distal bulb in *D. subpulchrella* to a sharper and more streamlined ovipositor in *D. suzukii*, explain their differential ability to pierce the skin of berries and grapes. Another possible explanation is that *D. subpulchrella* has fewer modified bristles along the lateral margins of the ovipositor, likely resulting in lower ability to puncture fruit skins. Atallah et al. (2014) described the ovipositor bristles as modified, enlarged, and strongly pigmented structures, differentiating between lateral and marginal depending on their location in the distal portion of the ovipositor. In addition, they reported that the total number of modified bristles (lateral and marginal) did not differ between *D. suzukii* and *D. subpulchrella*. However, we counted lateral and marginal bristles in theirs and our own (data not shown) images and found that *D. suzukii* has not only more MLBs but also a greater total number. While the shape of the organ may play an important role in the ability of *D. suzukii* to access new ecological niches, we show that the number of MLBs could also influence the species' ability to pierce the skin of fruit. In particular, we showed that the number of MLBs is positively associated with the number of punctures in blueberry fruits, suggesting that it may contribute to *D. suzukii*'s oviposition ability.

Just as saws intended for harder materials typically have more teeth, a higher number of MLBs might help *D. suzukii* pierce tougher fruit skins, while fewer bristles could be better suited for softer fruits. Microscale laser surgery has previously been used to experimentally manipulate genital morphology in seed beetles (Hotzy et al. 2012), and a similar approach could be adapted to selectively reduce MLB number in *D. suzukii* without damaging the rest of the ovipositor. This would enable a more direct test of the causal role of MLB number in fruit‐penetrating ability. Future experiments could combine such manipulations with assays using fruits of varying firmness and incorporate other bristle characteristics—such as length—to better understand how ovipositor morphology and host plant traits interact to shape oviposition success.

The influence of rearing temperature on morphological structures of *D. suzukii* adults, including wings, thorax, and ovipositor, has been extensively documented (Fraimout et al. 2018; Clemente et al. 2018; Shearer et al. 2016). Specifically, larval development under colder conditions is slower and results in the emergence of a distinct winter morph, characterized by larger adult body size (Shearer et al. 2016; Wallingford et al. 2016). Consistent with these findings, our results demonstrate a significant impact of rearing temperature on the number of MLBs in three out of the four assessed lines (Figure 4). In all cases, flies reared at 17°C exhibited more modified bristles than those reared at 25°C. Interestingly, the lines exhibiting plasticity in MLB number are those inhabiting temperate regions, whereas the less plastic line derives from a region with extreme winter conditions. All in all, these results further emphasize the critical role of temperature in shaping phenotypic variation in *D. suzukii*.

Regarding bilateral asymmetry of MLB number, rearing temperature did not affect fluctuating asymmetry. Fluctuating asymmetry, a measure of developmental stability (Hasson et al. 2008; Benitez and Parra 2011), serves as an indicator of the robustness of developmental processes under varying environmental conditions. The absence of temperature‐induced effects on fluctuating asymmetry in our study suggests that ovipositor development remains equally stable across the two temperature regimes examined. These results align with previous research, showing that neither temperature nor diet significantly affects bilateral symmetry of the ovipositor shape (Clemente et al. 2018). Stability may stem from strong morphofunctional constraints imposed by the ovipositor's mechanical role, which is likely under intense selective pressures (Clemente et al. 2018). The number of MLBs and the shape of the ovipositor plates may be under comparable selective pressures to preserve ovipositor functionality. While other studies have demonstrated that ovipositor size exhibits varying plasticity across lines under different temperature conditions, it appears to be more limited relative to other structures, such as wings (Varón‐González et al. 2020). Such restricted variation highlights the phenotypic robustness of the ovipositor shape, likely reflecting a history of stabilizing selection (Varón‐González et al. 2020). Altogether, these findings highlight the evolutionary importance of maintaining ovipositor functionality under varying environmental conditions.

Our fruit‐based experiments support the hypothesis that the number of MLBs contributes to oviposition success in *D. suzukii*. Across the three independent oviposition trials involving females from different sources, we consistently observed a positive correlation between MLB number and the number of punctures made on blueberry skin (Figure 6). On average, each additional MLB was associated with an 18% increase in puncture count. When accounting for experimental conditions (i.e., line, rearing temperature or selection regime in the first‐, second‐ and third‐source experiments, respectively), MLB number remained a significant predictor in both the first and second source datasets, indicating that the relationship holds within lines and temperature treatments. In the third source experiment, however, neither MLB number nor the condition factor showed significant effects, likely due to strong collinearity between the two variables.

Interestingly, in the first‐ and second‐source experiments, the condition explanatory variable also had a significant effect on the number of punctures, suggesting that other factors besides the number of MLBs may also play a role. This is particularly clear in the first‐source experiment, where despite line C14 having, on average, 1.7 more MLBs than M1, it produced 1.44 fewer punctures. Since the C14 and M1 lines originate from geographically distant populations, population‐specific factors unrelated to the number of MLBs may also influence flies' ability to penetrate the fruit skin. In the second‐source experiment, females reared at 17°C had, on average, 2.7 more MLBs than those reared at 25°C and produced 4.34 more punctures. This difference far exceeds what would be expected if the number of MLBs is the sole predictor. Therefore, these findings suggest that rearing temperature or population of origin influences other morphological, physiological, or behavioral traits that, in conjunction with the number of MLBs, shape the flies' ability to penetrate fruit skin.

Since we did not measure additional morphological traits—such as ovipositor size or shape—in the individuals tested for piercing ability, it may be argued that MLB number serves as a proxy for other causative traits. We acknowledge that other factors may influence piercing performance independently of MLB number, act in conjunction with it, or even co‐vary due to shared developmental or genetic pathways. However, the fact that MLB number did not correlate with ovipositor length or width in a subset of individuals from the M1 line reared at 17°C or 25°C (Figure 8) suggests that the observed association between MLB number and puncture count is unlikely to be explained solely by ovipositor size. This strengthens the hypothesis that MLBs themselves contribute directly to fruit‐piercing ability.

Furthermore, our data show a strong positive correlation between the number of punctures made on blueberry skin and the number of eggs laid (Figure 7), indicating that increased puncturing is associated with higher oviposition. While this could suggest a potential link between MLB number and reproductive output, our study did not assess egg viability or larval emergence. Therefore, we cannot ascertain whether more punctures more eggs result in greater offspring production. Future research tracking offspring survival and performance would help clarify how oviposition success relates to fitness outcomes in *D. suzukii*.

As mentioned above, the hardness of fruit skin may plausibly influence *D. suzukii*'s piercing activity (Kinjo et al. 2013). This variation can occur not only across different host fruit species but also within the same fruit species as it ripens, since ripening generally softens the fruit skin and makes it more penetrable (e.g., Chen et al. 2015; Lee et al. 2016). To account for this in our assays, we classified each fruit used in the oviposition trials into three ripeness categories (low, intermediate or high) and evaluated ripeness as an explanatory variable. However, this analysis revealed no consistent effect of ripeness across the three experimental datasets, with two showing non‐significant results. In contrast, the number of MLBs remained a significant predictor of puncture count across all three datasets. These results suggest that MLB number is a more reliable predictor of piercing ability than fruit ripeness and that our conclusions are not strongly confounded by fruit condition. Still, future studies could further investigate how fruit traits—including mechanical resistance and epidermal properties—interact with ovipositor morphology to shape host use in *D. suzukii*.

In summary, our results show that the number of MLBs: (1) varies within and between populations, (2) is heritable, (3) responds to artificial selection, (4) is plastic to the rearing temperature, (5) correlates positively with the number of punctures produced by females during oviposition in blueberry fruits, and (6) does not appear to correlate with ovipositor size within the same line and rearing temperature condition. Together, these findings indicate that MLBs could play an important role in the ability of the agricultural pest *D. suzukii* to pierce the skin of blueberry fruits. Moreover, our study provides a foundation for future research aimed to unravel the genetic basis of variation in the number of MLBs, which will provide a deeper understanding of the potential for evolutionary responses to natural selection and environmental pressures in this pest species.

## Author Contributions

**Madelein Sara Micaela Ortiz:** conceptualization (equal), investigation (equal), methodology (equal), writing – original draft (equal), writing – review and editing (equal). **L. Gandini:** formal analysis (equal), methodology (equal). **M. C. Sabio:** methodology (equal). **L. E. Bennardo:** methodology (equal). **L. M. Matzkin:** resources (equal), writing – review and editing (equal). **E. Hasson:** conceptualization (equal), funding acquisition (equal), project administration (equal), supervision (equal), writing – original draft (equal). **J. Hurtado:** conceptualization (equal), funding acquisition (equal), project administration (equal), supervision (equal), writing – review and editing (equal).

## Conflicts of Interest

The authors declare no conflicts of interest.

## Data Availability

All datasets and scripts underlying our analyses are available at Zenodo (https://doi.org/10.5281/zenodo.16509537).
